# Exploration of Biomarkers of Food Intake in a Caribbean Hispanic Population

**DOI:** 10.1002/mnfr.70158

**Published:** 2025-07-21

**Authors:** Laurence D. Parnell, Liam E. Fouhy, Oladimeji Akinlawon, Chao‐Qiang Lai, Frederick Nusetor, José M. Ordovás, Kelsey M. Mangano, Katherine L. Tucker, Sabrina E. Noel

**Affiliations:** ^1^ Agricultural Research Service US Department of Agriculture JM‐US Department of Agriculture Human Nutrition Research Center on Aging at Tufts University Boston Massachusetts USA; ^2^ Department of Biomedical and Nutritional Sciences University of Massachusetts Lowell Massachusetts USA; ^3^ Center for Population Health Zuckerberg College of Health Sciences University of Massachusetts Lowell Massachusetts USA; ^4^ Biostatistics and Data Management Unit JM‐USDA Human Nutrition Research Center on Aging at Tufts University Boston Massachusetts USA; ^5^ Precision Nutrition Group JM‐USDA Human Nutrition Research Center on Aging at Tufts University Boston Massachusetts USA; ^6^ IMDEA‐Food Institute CEI UAM+CSIC Madrid Spain; ^7^ Department of Public Health University of Massachusetts Lowell Massachusetts USA

**Keywords:** biomarker, diet assessment, food intake, genetics, metabolomics

## Abstract

Valid measures of dietary intake are essential for health and nutrition research, but typical forms‐ or interview‐based measurements are susceptible to random and systematic errors. Although many biomarkers of food intake (BFIs) have been validated, we aimed to explore how food‐BFI relationships are affected by genetic and lifestyle factors among Caribbean Hispanic adults. Dietary, clinical, anthropometric, blood metabolomics, and genotype data from 782 Puerto Rican adults were available. Thirty‐one BFI‐food intake relationships were assessed using linear regression, including covariates based on significant covariate‐BFI associations (i.e., age, body weight, physical activity, and sex). We observed 12 known BFI‐food pairs that reached statistical significance, of which 11 remained significant after adjustment. Applying genome‐wide association tests of blood metabolites to BFI‐food pairs, genetic variants rs7078243 and rs62501664 were identified as modifying relationships with 3‐methylxanthine‐coffee and 3‐methylhistidine‐poultry, respectively. Eleven validated biomarker‐food intake pairs remained statistically significant after adjusting for covariates. Identification of genotype‐BFI associations accentuates that the implementation of certain BFIs will depend on common genetic differences.

## Graphical abstract

Validated BFIs show significant associations with food intake in an observational setting, and those associations were often modulated by non‐dietary factors. The identification of genotype‐BFI associations implies that the utility of a BFI could depend on common genetic differences.

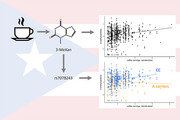

## 1 Introduction

Data on dietary intake, which is central to research on diet‐related health outcomes, traditionally has been collected using self‐reported food intake from food frequency questionnaires (FFQ), 24‐h dietary recalls, or dietary records. These dietary assessment tools are subject to systematic and random errors, such as recall bias, under‐ or over‐reporting, poor estimation of portion size, and measurement error [1] that affect the validity of dietary data, particularly among older adults [2]. Biomarkers of food intake (BFIs) provide a more objective method of obtaining dietary data compared with self‐reported assessments and can provide valuable information on variability in biological responses to food across individuals (e.g., differences in metabolism between individuals) [3]. Many validated BFIs are qualitative, a type of binary (true/false) assessment indicating intake near the time a clinical specimen was obtained [4]. Thus, implementing BFIs in population‐based studies has the potential to add elements of precision to understanding diet‐health relationships. This is a useful aspect of precision nutrition as it seeks to determine more accurately what a person consumes and the relationship between dietary intake and health outcomes. However, challenges remain, including representation by BFIs of a wide number of foods and culturally relevant foods in which rigorous BFI discovery efforts have not begun.

There are several factors regarding BFIs that must be considered [5]. Although metabolite concentrations can correlate with food consumption and those correlations permit ranking of individuals by food intake, differences in metabolism and other characteristics can alter those rankings. Additionally, both the half‐life of a biomarker, anywhere from 4 h to several days, and habitual or chance consumption of food just prior to sample collection can affect the BFI‐food intake association. Lastly, the use of combinations of biomarkers may increase the accuracy and reproducibility of estimates of food intake [4, 5, 6].

Various factors have the potential to change, possibly weaken, the relationship between food intake and BFI concentration in blood or urine. These include the food matrix, gut transit time, genetic variation, and lifestyle factors such as physical activity [4, 5, 7, 8]. Genomic data can be incorporated with BFIs to determine the influence of genetic variants and gene‐diet interactions on diet‐related health outcomes such as diabetes and cardiovascular disease. A metabolite‐quantitative trait locus (mQTL) is a genetic marker that associates with metabolite abundances in a given sample, or a genotype‐phenotype association where the metabolite abundance is the phenotype. Because urinary and blood BFI concentrations represent both food intake and protein activity, such as absorption, transport, and enzymatic processes of that BFI metabolite [4], genetic variation in the genes encoding these proteins may modulate the food intake‐BFI relationship. For example, a study involving elite athletes identified five loci as significantly associated with stachydrine levels, including *NUDT4* gene variant rs10859481 [9]. Further, proline betaine, also known as stachydrine and a BFI for citrus intake, has been found to be higher in those with elevated inflammasome activity [10]. Furthermore, this metabolite has been associated with a 0.32‐fold change after a 4‐day 51‐km cross‐country skiing arctic military training exercise [11]. Such observations exemplify the multitude of attributes any BFI exhibits and prompts investigations on how these factors can affect the food intake‐BFI association.

Although BFIs support a way to attain precision nutrition in a retrospective manner, precision nutrition also encompasses building predictive models for future health. These models, built with machine learning (ML) tools, use as input genomics, epigenomics, and dietary data, for example [12], and can be extended to include metabolomics and other omics datasets. Inclusion of dietary data and metabolomics in the modeling necessitates acknowledgment of the food intake‐BFI catalogs, as it may require weighting certain input variables in the ML models. The primary aims of this study were to examine associations between BFIs and related foods, identify genetic variations associated with specific metabolite biomarkers, and determine the impact of genotype on BFI‐food intake correlations. Findings from this study may contribute to future personalized nutrition recommendations and interventions tailored to individual genetic makeup and metabolic profile for disease prevention and management.

## 2 Experimental Section

### 2.1 Study Population

This study included data for 782 participants from the Boston Puerto Rican Health Study (BPRHS). Puerto Rican adults aged 45–75 years were recruited from the Greater Boston area through door‐to‐door enumeration and community‐engaged activities [13]. Of the 1504 participants who completed the baseline interview, 817 plasma samples were sent to Metabolon, Inc. (Morrisville, NC, USA) for small‐molecule analysis, of which 782 had complete data. All participants provided written informed consent. Details of data collection procedures have been reported in previous publications, and the methods described in this study partly reproduce their wording [14, 15]. The study was approved by the Institutional Review Boards at Tufts University (6629, 6763) and the University of Massachusetts Lowell (13‐109‐TUC‐XPD) and adhered to the ethical principles of the Helsinki Declaration of 1975 as revised in 1983.

### 2.2 Dietary Assessment

A FFQ adapted and validated for use in this population was used to collect dietary intake data [16]. The FFQ was built using the National Cancer Institute/Block FFQ and modified using dietary recalls from the Hispanic Health and Nutrition Examination Survey for Puerto Rican adults to include foods and portion sizes that represent a typical Puerto Rican Diet. The FFQ has been shown to be a better estimator of dietary intake in this Hispanic population compared with the original Block questionnaire [16]. Serving sizes for individual food items were determined directly from the FFQ using common portions or predetermined units (e.g., one apple, one slice of bread, etc.). For mixed dishes, ingredients were disaggregated into gram amounts contributing to each overall recipe, and servings were determined from gram amounts. Mean daily nutrient intakes were calculated using the Nutrient Data System for Research software, version 2007 (Nutrition Coordinating Center, University of Minnesota, Minneapolis, MN, USA). Dietary data were available for all 782 participants for whom anthropometric, genotype, and metabolomics data were also available.

### 2.3 Metabolomics

Metabolic profiling of plasma samples was performed by Metabolon, Inc. (Morrisville, NC, USA), as described elsewhere [17]. Briefly, frozen plasma samples were shipped on dry ice and stored at −80°C until analysis. After methanol precipitation of proteins, metabolomic analysis employed ultrahigh‐performance liquid chromatography‐tandem mass spectroscopy. Individual metabolites, first identified by reference to a library of over 4500 purified standards for retention time/index, mass‐to‐charge ratio, and chromatographic data, were quantified by estimating the AUC of the peaks. The data were normalized in two ways. First, the metabolite data were normalized for batch effects by and according to the provider's method [18], followed by log‐transformation to obtain normally distributed values. The median relative standard deviation for internal standards (a measure of instrument variability akin to the coefficient of variation) was 5%. After normalization across samples, 526 targeted metabolites passed quality control, and 20 of these 526 metabolites were noted as validated BFIs [4].

### 2.4 Selected BFIs for Analyses

A recent systematic review highlighted validated BFIs for several consumed foods [4]. Based on this review, we selected 20 BFI metabolites and related foods. Given unique cultural components of Puerto Rican dietary intake, additional foods were linked to specific BFIs (Table 1). For example, S‐allylcysteine was identified as a BFI for both garlic and sofrito, which is a vegetable base made of onions, garlic, peppers, tomatoes, and herbs used in Puerto Rican mixed dishes and has been shown to have positive health effects [15, 19]. Proline betaine is a BFI for orange [20], and we considered intake of orange fruit and juice, separately and combined. We also assigned S‐methyl‐L‐cysteine, described as a BFI for white beans or dry beans [4], as a potential indicator for all beans, dried or canned. BFIs used in this study, including BFI, related food, PubChem database identifier, and notes regarding analysis are reported in Table 1. Added to the list are three other metabolites including (S)‐3‐methyl‐2‐oxopentanoate, 2‐oxoglutarate, and the ratio of isoleucine to glutamate, which are related to the biochemical reactions catalyzed by BCAT1 and BCAT2 (branched chain amino acid transaminases). These two amino acids are known BFIs for cheese intake.

**TABLE 1:** Biomarkers of reported food intake examined in the Boston Puerto Rican Health Study.

Food/food group	BFI	PubChem identifier	Modification for analysis
Cheese	Glutamate	611	
Cheese	Isoleucine	6306	
Cheese	(S)‐3‐methyl‐2‐oxopentanoate	4 298 878	See note
Cheese	2‐oxoglutarate	51	See note
Coffee	3‐methylxanthine	70 639	
Coffee	Paraxanthine	4687	
Coffee	Trigonelline	5570	
Fish and fatty fish	3‐carboxy‐4‐methyl‐5‐propyl‐2‐furanpropionic acid	123 979	
Fish and fatty fish	Docosahexaenoic acid	445 580	
Fish and fatty fish	Eicosapentaenoic acid	446 284	
Fish and fatty fish	Trimethylamine N‐oxide	1145	
Grains, all	Hippuric acid	464	
Grains, refined	Hippuric acid	464	
Grains, whole	Hippuric acid	464	
Grains, whole	Trigonelline	5570	
Garlic	S‐allylcysteine	9 793 905; 98 280	
Sofrito	S‐allylcysteine	9 793 905; 98 280	
Grape	Tartaric acid	875	
Wine, red	Tartaric acid	875	
Milk	D‐galactonic acid	128 869	All dairy milk
Milk	Gluconic acid	10 690	All dairy milk
Orange, fruit + juice	Proline betaine	115 244	
Orange, fruit	Proline betaine	115 244	
Orange, juice	Proline betaine	115 244	
Poultry (chicken)	3‐methylhistidine	64 969	
Pulses	Trigonelline	5570	
Pulses	S‐methyl‐L‐cysteine	24 417	
White beans or dry beans	S‐methyl‐L‐cysteine	24 417	All beans: dried and canned
Terrestrial meat	Beta‐alanine	239	Chicken, cow, pig, veal, deer, organ meats, morcilla
Vegetables	Trigonelline	5570	

*Note*: Validated biomarkers selected based on a recent systematic review [4].

Not a validated BFI for cheese, but a constituent of the transamination reaction interconverting isoleucine and glutamate, catalyzed by BCAT1/BCAT2.

### 2.5 Genotype Data

Genotyping was performed using the Affymetrix Axiom Genome‐Wide LAT 1 Array as described [21]. Of 817 810 genotyped single‐nucleotide polymorphisms (SNPs), 712 197 autosomal SNPs passed quality control and met criteria of call rate ≥97%, MAF ≥1%, and *p* value for Hardy‐Weinberg equilibrium (HWE) ≥10^−6^. To control for population structure, principal component analysis (PCA) was performed [22] in SVS (GoldenHelix Inc., Bozeman, MT, USA) using 50 704 SNPs, which were selected based on the following criteria: call rate >97%, MAF ≥5%, pairwise linkage disequilibrium *R*‐squared ≤0.1, HWE P ≥10^−6^. The first principal component (PCA1) represents the major proportion of variation in population structure and hence was selected based on the scree plot to adjust for population structure in all regression models [22].

### 2.6 Covariates

Age (years) and sex (male and female) were quantified through a questionnaire [13]. A physical activity score was derived from a modified Paffenbarger questionnaire of the Harvard Alumni Activity Survey [13]. Smoking status was classified as never, past, or current, and alcohol consumption as none, moderate (≤1 drink/d for females or ≤2/d for males), or heavy (>1 for females or >2 for males) based on self‐reported questionnaire. Height (cm) and weight (kg) were measured following standard procedures in duplicate, and the mean of the two was used for each [23].

### 2.7 Statistics

Analyses were performed in R (version 4.2.2) [24] and RStudio (version 2023.03.1+446) (Posit Software, Boston, MA, USA). In this section, references to packages and relevant specific functions take the following format: package::function (settings). Settings that deviate from the default are noted in parentheses.

#### 2.7.1 Exploratory Data Analysis (EDA)

Where appropriate (e.g., servings of each food/food group, but not for sex), data were normalized to the mean by Z‐score. To learn about the structure in the data and to identify any factors that are significantly associated with one another in ways that could alter a simple view of the BFI‐food intake relationship, we explored the data. This exploration of factors relating separately to BFIs and dietary intakes was done with generalized linear models as rstats::glm (family = gaussian). Results of these analyses justify the use of specific covariates. Heteroscedasticity was checked with the Breusch‐Pagan test, car::ncvTest. If age, body weight, physical activity, or sex were associated with either a specific BFI metabolite or intake of certain foods, then those factors were included as covariates in multivariable regression models. Similarly, covariates that exhibited significant heteroscedasticity were also included in models. A schematic illustrating datasets used and the analyses performed is presented in Figure S1.

#### 2.7.2 Association Between BFIs and Foods

Multivariable linear regression models were examined with BFI as dependent outcomes, food intake (servings/day) as a predictor, and covariates as identified above, rstats::lm. The primary covariates included age (years), body weight (kg) as a proxy for blood volume [25], physical activity (as a continuous physical activity score), and sex (male/female). Two models were tested: Model 1 was outcome and food intake without covariates; Model 2 added the covariates for each BFI discovered in the EDA by either linear regression or heteroscedasticity.

#### 2.7.3 Genome‐Wide Association With BFI Abundances

To identify genetic variants associated with relative abundances of metabolites having a BFI attribute, we performed a genome‐wide association study (GWAS) of 712 197 SNPs for each of the 20 BFI metabolites using mixed linear regression models with each metabolite as outcome and SNP genotype as predictor, adjusting for sex, age, smoking, alcohol consumption, and population substructure (PCA1). A dominant effect of the minor allele was evaluated for SNPs with MAF <0.05. Each GWAS was implemented using GoldenHelix SNP & Variation Suite 8.9.1. Multiple testing was corrected based on the Bonferroni test, with genome‐wide significance set at *p* ≤ 5×10^−8^, derived from 0.05/10^6^. Secondarily, we also consider a nominal GWAS significance threshold of 10^−5^, as experience has shown that modest associations may reach statistical significance after adjusting for covariates or when included in the model as a gene by diet or gene by environment interaction [26].

The extent of the mQTL results (10 associations for four different BFI metabolites plus 241 other associations with *p* values above genome‐wide significance) suggests widespread genetic influence on blood metabolites in this population [9, 10, 11, 27, 28]. Thus, we first tested the 10 genome‐wide significant associations for effects on how well the BFI represents food intake in the BPRHS population. Fourteen such analyses were performed with four SNP genotypes for tartaric acid—rs17230437, rs36089417, rs115208179, and rs8014077–analyzed twice because of potential relationships with intake of grapes and red wine. For each SNP, the population was split into two subsets by genotype: major allele homozygotes and minor allele carriers as this matches the dominant model applied in the mQTL analysis. Linear regression analysis was performed to characterize the BFI‐food intake relationship for the BFI within each genotype group.

## 3 Results

### 3.1 Characteristics of Study Participants

Data from 782 participants were available from the BPRHS, whose characteristics have been reported in other studies of diet and diet quality [29] and metabolomics [15]. A total of 555 of 782 participants (71.0%) were female, with a mean age of 57.3 years, and the mean weight of females was 79.2 kg and of males 84.9 kg (Table 2).

**TABLE 2:** Characteristics of the Boston Puerto Rican Health Study population.

Sex: *n* (%)	Female: 555 (71.0%)	Male: 227 (29.0%)
**Body weight, kg: mean (IQR)**	79.2 (66.8–88.6)	84.9 (74.5–95.5)
**Waist, cm: mean (IQR)**	102 (91.0–112)	103 (94.0–110)
**Body mass index, kg/m^2^: mean (IQR)**	32.9 (28.1–36.7)	30.1 (26.7–33.5)
**Age, y: mean (IQR)**	57.3 (51–63)	
**Smoker: never, past, current (%)**	363 (46.4%), 238 (30.4%), 180 (23.0%)	
**Alcohol drinker: never, past, current (%)**	222 (28.4%), 236 (30.2%), 321 (41.0%)	
**Physical activity: mean (IQR)**	31.6 (28.2–33.3)	

### 3.2 Associations Between BFIs and Related Foods and Food Groups

Twelve significant associations between intake of specific foods and food groups and BFI metabolites were observed, 11 of which remained statistically significant after adjusting for covariates (Table 3). Specifically, associations between 3‐methylxanthine and coffee, docosahexaenoic acid (DHA) and fish, and proline betaine and orange juice/fruit were noted (all *p* < 8.1×10^−6^). BFI S‐allylcysteine associated significantly with garlic intake (*p* = 0.0498). The association between 3‐methyl‐2‐oxopentanoate and cheese intake, however, was attenuated after adjustment for covariates. The association between terrestrial meat intake and blood abundance of beta‐alanine did not reach statistical significance (*p* = 0.31), but this is likely because urinary metabolites were not available in this population [4]. Associations between food intake and corresponding BFIs are dependent on BFI kinetics coupled with the frequency of intake [4]. It is possible that some BFI‐food associations did not reach statistical significance in this study because dietary intake was obtained by FFQ, which assesses consumption over the past year, and specific timing of intake is not known.

**TABLE 3:** Relationships between biomarkers of food intake and their related foods in the Boston Puerto Rican Health Study.

BFI	Food	*p* value (Model 1)	*p* value (Model 2)	Model 2 covariates
Glutamate	Cheese	0.418	0.097	Age;sex;weight
Isoleucine	Cheese	0.623	0.909	Age;sex;weight
Isoleucine: glutamate ratio	Cheese	0.271	0.109	Age;pa;sex;weight
(S)‐3‐methyl‐2‐oxopentanoate	Cheese	0.034	0.169	Age;sex;weight
2‐oxoglutarate	Cheese	0.807	0.852	Pa;sex;weight
3‐methylxanthine	Coffee	1.18E‐06	1.35E‐06	Weight
Paraxanthine	Coffee	2.47E‐08	8.12E‐08	Pa;weight
Trigonelline	Coffee	1.51E‐14	5.86E‐16	Age;pa;weight
3‐carboxy‐4‐methyl‐5‐propyl‐2‐furanpropionic acid	Fish and fatty fish	8.26E‐12	1.06E‐12	Age
Docosahexaenoic acid	Fish and fatty fish	2.07E‐05	8.09E‐06	Age
Eicosapentaenoic acid	Fish and fatty fish	2.58E‐03	5.02E‐03	Age;pa;weight
Trimethylamine N‐oxide	Fish and fatty fish	0.0706	0.605	Age;pa
Hippuric acid	Grains, all	0.164	0.343	Age
Hippuric acid	Grains, refined	0.164	0.302	Age
Hippuric acid	Grains, whole	0.436	0.444	Age
Trigonelline	Grains, whole	0.429	0.580	Age;pa;weight
S‐allylcysteine	Garlic	0.0843	0.0498	Sex
S‐allylcysteine	Sofrito	0.12	0.813	Sex
Tartaric acid	Grape	0.612	0.883	Age;pa;sex
Tartaric acid	Wine, red	0.567	0.437	Age;pa;sex
D‐galactonic acid	Milk	0.519	0.596	Sex
Gluconic acid	Milk	0.896	0.653	Age;pa;sex;weight
Proline betaine	Orange, fruit + juice	2.67E‐09	9.23E‐10	Age
Proline betaine	Orange, fruit	3.28E‐10	1.17E‐10	Age
Proline betaine	Orange, juice	2.54E‐08	8.71E‐09	Age
3‐methylhistidine	Poultry (chicken)	0.018	0.0113	Age
Trigonelline	Pulses	0.133	0.177	Age;pa;weight
S‐methyl‐L‐cysteine	Pulses	0.356	0.379	Age;sex
S‐methyl‐L‐cysteine	White beans or dry beans	3.63E‐04	3.66E‐03	Age;sex
Beta‐alanine	Terrestrial meat	0.309	0.309	None
Trigonelline	Vegetables	0.62	0.456	Age;pa;weight

*Note*: The isoleucine: glutamate ratio, (S)‐3‐methyl‐2‐oxopentanoate, and 2‐oxoglutarate are not validated BFIs, but relevant to BCAT1/BCAT2 enzymatic activity.

Model 1: no covariates included.

Model 2: covariates added based on significant association with BFI abundances in blood and heteroscedasticity.

*p* is for the BFI food intake association.

### 3.3 Associations Between Genotype and BFI Metabolites

Genotype or DNA methylation patterns have been associated with metabolites in blood and urine [9, 17, 27]. In a dominant model adjusted for population structure, sex, age, physical activity, educational attainment, total energy intake, smoking, and alcohol consumption, 251 nominal associations (249 unique SNPs, *p* < 10^−5^) were observed. At genome‐wide significance using a strict *p* value threshold of 5×10^−8^, 10 associations with four BFI metabolites (3‐methylxanthine, gluconic acid, isoleucine, and tartaric acid) were statistically significant (Table 4). The most significant of the genetic association signals was tartaric acid at SNP rs17230437 (*p* = 2.55×10^−15^), mapping to an intron of *CCDC7*, coiled‐coil domain containing 7. Other associations included SNPs rs7078243 in gene *KIF11* (kinesin family member 11) which was associated with 3‐methylxanthine (*p* = 3.20×10^−8^), rs17622614 within gene *WASL* (WASP like actin nucleation promoting factor) with gluconic acid (*p* = 2.49×10^−14^), and rs62441697 within long intergenic non‐protein coding RNA 1162, *LINC01162*, associating with isoleucine (*p* = 1.50×10^−8^).

**TABLE 4:** Genome‐wide associations of metabolite biomarkers of food intake in the Boston Puerto Rican Health Study.

BFI	SNP	Effect allele	Chr	Position	nearest gene	*p* value	Predictor beta	Predictor beta SE
3‐methylxanthine	rs7078243	A	10	92654506	*KIF11*	3.20E‐08	0.454	0.081
Gluconic Acid	rs17622614	C	7	123686281	*WASL*	2.49E‐14	−1.22	0.157
Gluconic Acid	rs76499143	T	9	76196647	*PCSK5*	2.36E‐08	−0.38	0.067
Gluconic Acid	rs79286023	T	7	98572380	*NPTX2*	3.46E‐08	−0.793	0.142
Gluconic Acid	rs17596997	T	4	166380587	*SPOCK3*	3.56E‐08	−0.419	0.075
Isoleucine	rs62441697	T	7	21012358	*LINC01162*	1.50E‐08	0.196	0.034
Tartaric Acid	rs17230437	T	10	32581169	*CCDC7*	2.55E‐15	−0.821	0.102
Tartaric Acid	rs36089417	C	5	33994939	*AMACR*	3.52E‐15	−0.772	0.096
Tartaric Acid	rs115208179	T	1	223128813	*TLR5*	3.55E‐14	−0.773	0.1
Tartaric Acid	rs8014077	A	14	58372621	*ARID4A*	1.13E‐08	−0.538	0.093
2‐oxoglutarate	rs17622614	C	7	123686281	*WASL*	1.10E‐15	−1.24	0.152
2‐oxoglutarate	rs77913521	C	6	150651018	*PLEKHG1*	1.10E‐09	−0.644	0.104
2‐oxoglutarate	rs79338118	A	5	160889958	*ATP10B*	1.75E‐09	−0.947	0.155
2‐oxoglutarate	rs111518260	C	1	246194777	*SMYD3*	4.08E‐09	−0.521	0.088
2‐oxoglutarate	rs28753943	T	14	86927409	*LINC01148*	5.71E‐09	−0.603	0.102
2‐oxoglutarate	rs11144614	C	9	68977006	*PIP5K1B*	8.21E‐09	−0.452	0.078
2‐oxoglutarate	rs117833917	C	19	42214074	*DEDD2*	9.27E‐09	−0.611	0.105
2‐oxoglutarate	rs76758982	G	7	123716586	*WASL*	2.06E‐08	−0.562	0.099
2‐oxoglutarate	rs6539766	G	12	83656993	*TMTC2, LOC105369874*	2.26E‐08	−0.589	0.104
2‐oxoglutarate	rs76259438	A	10	113799872	*PLEKHS1*	3.48E‐08	−0.624	0.112
2‐oxoglutarate	rs113972487	G	12	20369279	*PDE3A*	3.59E‐08	−0.61	0.11

*Note*: Genome coordinates are from build GRCh38.p14.

### 3.4 Special BFI Cases—Ratios and Reactions Relevant to BCAT1/BCAT2 Enzymatic Activity

To enhance identification of genetic factors associated with metabolites, a ratio of two metabolites can be used as it increases statistical power to detect relevant loci [30], particularly when common enzymatic processes link two metabolites. Among the BFI metabolites included in this study, the bidirectional reaction catalyzed by BCAT1 and BCAT2, isoleucine + (S)‐3‐methyl‐2‐oxopentanoate, converts to glutamate + 2‐oxoglutarate. Thus, the ratio of isoleucine to glutamate, both BFIs for cheese consumption, as well as (S)‐3‐methyl‐2‐oxopentanoate and 2‐oxoglutarate, individually, were examined. The mQTL analysis identified 11 distinct genomic regions associated with 2‐oxoglutarate at genome‐wide significance (Table 4). The top signal was noted with variant rs17622414 in *WASL* (*p* = 1.10×10^−15^); however, no statistically significant associations were noted for the ratio of isoleucine to glutamate or 3‐methyl‐2‐oxopentanoate.

### 3.5 Impact of Genotype on BFI‐Food Associations

**FIGURE 1:**
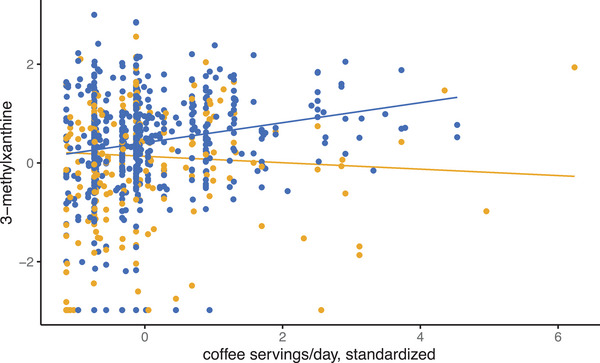
SNP rs7078243 genotype effect on the correlation between coffee intake and blood 3‐methylxanthine. Participants who are homozygous for the major allele at rs7078243 have genotype C/C and show a positive relationship between coffee intake (servings per day) and relative abundance in blood of 3‐methylxanthine, a caffeine metabolite. Those who are carriers of the A minor allele show the opposite correlation. The A minor allele has an allele frequency of 0.441. Blue represents major allele homozygotes, while orange represents minor allele carriers, with the respective regression trend lines drawn.

**FIGURE 2:**
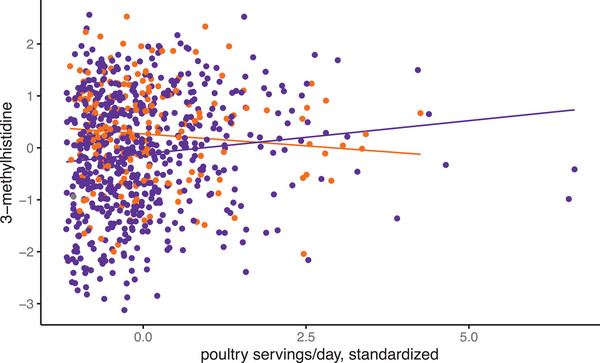
SNP rs62501664 genotype effect on the correlation between poultry intake and blood 3‐methylhistidine. Participants who are homozygous A/A for the major allele at rs62501664 show a positive relationship between poultry intake (servings per day) and relative abundance in blood of 3‐methylhistidine. Those who are carriers of the G minor allele show the opposite correlation. The minor allele G occurs in this population with a frequency of 0.132. Purple represents major allele homozygotes, while orange represents minor allele carriers, with the respective regression trend lines shown.

For major allele homozygotes of rs7078243, the correlation between coffee intake and blood 3‐methylxanthine was significant (*p* = 3.42×10^−6^, Figure 1, blue data points). None of the other associations reached statistical significance (Table 5). Interestingly, results of assessing the impact of genotype on BFI‐food intake associations (Table 5) showed stronger correlation with the BFI‐food intake relationships (Table 3) than the mQTL GWAS results (Table 4). This prompted an examination of 3‐methylhistidine as a BFI for poultry intake as an example of such observations. The BFI‐food relationship was marginally significant (*p* = 0.018, Table 3), and SNP rs62501664 was the most significant mQTL for 3‐methylhistidine (*p* = 1.22×10^−7^, data not shown), but just below the strict genome‐wide significance threshold. With the population split by genotype, as above, followed by characterization of the BFI‐food relationship, we observed a significant genotype effect. For major allele homozygotes of rs62501664, the relationship between poultry intake and relative abundance in blood of 3‐methylhistidine was moderately significant (*p* = 6.16×10^−3^, Figure 2, purple data points). For both rs7078243 and rs62501664, carriers of the minor allele did not attain significance for the BFI‐food intake correlation: for rs7078243 *p* = 0.056 and for rs62501664 *p* > 0.2. We observed another 112 nominal associations involving a validated BFI that are statistically significant with respect to intake of its cognate food in BPRHS. Together, these results imply that genotype effects are likely widespread, requiring in‐depth genome‐wide analyses.

**TABLE 5:** Impact of genotype on BFI‐food intake correlations in the Boston Puerto Rican Health Study.

SNP	BFI	Food	*p food‐BFI*, model 1	*p SNP‐BFI*, GWAS	*p food‐BFI* for genotype 1	*p food‐BFI* for genotype 2
rs7078243	3‐methylxanthine	Coffee	1.18E‐06	3.20E‐08	3.42E‐06	0.056
rs17622614	Gluconic acid	Milk	0.896	2.49E‐14	0.635	0.732
rs76499143	Gluconic acid	Milk	0.896	2.36E‐08	0.636	0.607
rs79286023	Gluconic acid	Milk	0.896	3.46E‐08	0.660	0.426
rs17596997	Gluconic acid	Milk	0.896	3.56E‐08	0.854	0.794
rs62441697	Isoleucine	Cheese	0.623	1.50E‐08	0.621	0.685
rs17230437	Tartaric acid	Grape	0.612	2.55E‐15	0.351	0.098
rs36089417	Tartaric acid	Grape	0.612	3.52E‐15	0.557	0.230
rs115208179	Tartaric acid	Grape	0.612	3.55E‐14	0.539	0.233
rs8014077	Tartaric acid	Grape	0.612	1.13E‐08	0.709	0.448
rs17230437	Tartaric acid	Wine, red	0.567	2.55E‐15	0.946	0.313
rs36089417	Tartaric acid	Wine, red	0.567	3.52E‐15	0.937	0.239
rs115208179	Tartaric acid	Wine, red	0.567	3.55E‐14	0.938	0.344
rs8014077	Tartaric acid	Wine, red	0.567	1.13E‐08	0.664	0.704

*Note*: genotype 1 = homozygous major allele; genotype 2 = minor allele carriers.

*p food‐BFI*, Model 1 ‐ taken from Table 3.

*p SNP‐BFI*, GWAS—taken from Table 4.

## 4 Discussion

Data from the BPRHS were used to examine relationships between blood BFIs and food consumption. Our results identified 11 statistically significant associations between BFI metabolites and related foods after adjusting for covariates, including age, weight, physical activity, and sex. We also examined the effect of genotype on the relationship between BFI‐food intake, focusing on two relationships with different *p* values –0.018 and 1.18×10^−6^. The two BFI‐food relationships identified were not significant for carriers of the minor allele of variants but were for the homozygous major allele. Our findings of genotype affecting BFI‐food intake relationships, combined with the identification of over 100 loci that were nominally (*p* < 10^−5^) associated with metabolites, highlight the importance of considering genotype in evaluating food‐BFI relationships, as others have proposed [4, 8].

A total of 10 SNPs for four BFIs, including 3‐methylxanthine, gluconic acid, isoleucine, and tartaric acid, were identified. These findings suggest that individuals with a specific genotype may present with different BFI‐food intake relationships than what is seen with the overall population, such that individuals with discordant BFI‐food intake data might carry a specific allele identifiable by GWAS. Moreover, enzyme, metabolism, and transport kinetics involving the BFI are also relevant [4], especially genetic variation. Variation in the sequence of genes encoding proteins functioning in these processes has been associated with components of metabolic syndrome (e.g., glutamate [31]) and may modulate the food‐BFI relationship. This was further examined using data on two genetic variants from our mQTL analysis to identify the nature of genotype‐specific BFI‐food intake relationships. One SNP‐BFI‐food intake with borderline statistical significance for both BFI and mQTL—rs62501664, 3‐methylhistidine, and poultry intake—and a second set that showed robust significance—rs7078243, 3‐methylxanthine, and coffee intake—were selected and examined by genotype to identify potential allele‐specific differences. In both cases, individuals homozygous for the major allele demonstrated that the BFI was associated with the respective food intake, but associations were not significant in carriers of the minor allele. In the mQTL analysis, for both SNPs, minor allele carriers had positive predictor beta values, indicating an increased amount of metabolite. Additional work is needed to determine how this relates to the significant BFI‐food relationship among the major allele homozygotes. In short, genotypes and their effects on BFIs should be considered.

The mQTL GWAS identified 10 genotype‐metabolite associations for validated BFIs at a genome‐wide significance level of *p* < 5×10^−8^. Apart from rs7078243 for 3‐methyxanthine, described above, nine other metabolites with significant mQTL signals (gluconic acid [milk], isoleucine [cheese], and tartaric acid [grape]) were not associated with foods when genotype was considered. Thus, only one of 10 mQTL loci supports a genotype effect on the BFI‐food relationship. However, the mQTL results contain 114 loci at nominal *p* value (<10^−5^) for BFIs with significant BFI‐food relationships. Interestingly, this suggests that the moderate genetic effect on BFI metabolite abundance may reach statistical significance when appropriate covariates are considered. For example, variant rs62501664, which modifies the relationship between poultry intake and the BFI 3‐methylhistidine, has a minor allele frequency (MAF) in BPRHS of 13%. Furthermore, while many genetic variants were identified with low MAF (between 0.5% and 5%) in this population, 60 others have been observed in various populations with MAFs above 20%. When comparing across global populations, 36% of these SNPs exhibit large (>20%) differences in their MAFs, implying a strong likelihood for environmental effects. Additionally, the strength of the genotype‐phenotype association partly depends on phenotype variance, which can differ across populations and blood and urine metabolomics data. Our findings emphasize that genetic diversity must be considered carefully when applying BFIs across different populations.

Considering the large differences in effect allele frequencies, our findings suggest that numerous genetic factors may affect BFI‐food intake relationships, and that established BFIs most likely will show some modification of the relationship with food intake in populations with different genetic ancestries. Overall, there appears to be complex relationships between food intake, BFIs, genotype and various demographic, lifestyle and anthropometric factors. Therefore, characterization of these relationships will require the development of specialized statistical models. We posit that efficient incorporation of genotypes into BFI‐food analyses begins with validated BFIs that associate significantly with foods, followed by addition of the genotype data from the population under study.

The current study has several strengths and limitations. Strengths include inclusion of a historically underrepresented population in health research; use of a culturally tailored and validated FFQ; consideration of covariates that could alter the BFI‐food associations; and rich genotypes, metabolomics and dietary data. Results supports the identification of common genetic variants that exert influence on the BFI‐food associations. The current work was limited by a modest‐sized sample restricted mainly to a defined age range. Metabolomics data affiliated with this population were limited to 20 BFIs in blood that related to intakes of 19 individual foods and food groups although these foods represent the five major food groups that constitute the USDA's Dietary Guidelines for Americans [32]. No urinary metabolomics data were available to assess urinary BFIs. Some dietary data did not match exactly to the food or food group in the initial BFI description (e.g., all dried and canned beans in place of white beans or dried beans), and in other instances, culturally relevant information demanded that an appropriate substitute food be considered for a given BFI. Furthermore, time‐based factors alter the BFI‐food intake relationship, meaning that blood samples may become disconnected in time from the FFQ responses for habitual dietary intakes. Without information on time since consumption, some validated BFIs with more sensitive kinetics might not reach statistical significance, especially when factoring in population‐specific allele frequencies. Lastly, replication of the genetic association results should be tested in other similar populations. The limitations described above highlight the need for carefully characterized BFIs prior to implementation in population studies [5] and suggest a need for collaborative research in determining BFIs and quantifying dietary intake from dietary assessment tools, work which could be supported in part by the Periodic Table of Foods Initiative [33].

## 5 Conclusion

In summary, an exploration of BFIs in a deeply phenotyped Caribbean Hispanic population identified 11 BFI‐food intake pairs and ten different genetic variants associated with four BFI blood metabolites. A survey to identify genotype‐specific BFIs based on two genetic variants identified by metabolite‐GWAS suggests that genotype is an important factor in the BFI‐food intake relationship and that more research in this area is needed. Future research, including BFIs, should consider covariates and genetic variants known to alter their appropriate relevance as an indicator of food intake.

## Disclosure

The USDA had no part in the design of this project, the collection, analysis, and interpretation of data nor in composing the manuscript. Mention of trade names or commercial products is solely for providing specific information and does not imply recommendation or endorsement by the USDA. The USDA is an equal opportunity provider and employer.

## Conflicts of Interest

The authors declare no conflicts of interest.

## Peer Review

The peer review history for this article is available at https://publons.com/publon/10.1002/mnfr.70158.

## Supporting information



[**Supporting File 1**: mnfr70158‐sup‐0001‐SuppMat.docx

## Data Availability

Owing to privacy and ethical considerations, the data used in this study are available upon reasonable request and review.
