# Supplementary material for: Exploration of Biomarkers of Food Intake in a Caribbean Hispanic Population
Source: Mol Nutr Food Res. 2025 Jul 21;69(20):e70158. doi: 10.1002/mnfr.70158 (PMC12538526; doi:10.1002/mnfr.70158)
Supplement: Supplementary file 1 — [Supporting File 1: mnfr70158‐sup‐0001‐SuppMat.docx [file MNFR-69-e70158-s001.docx]

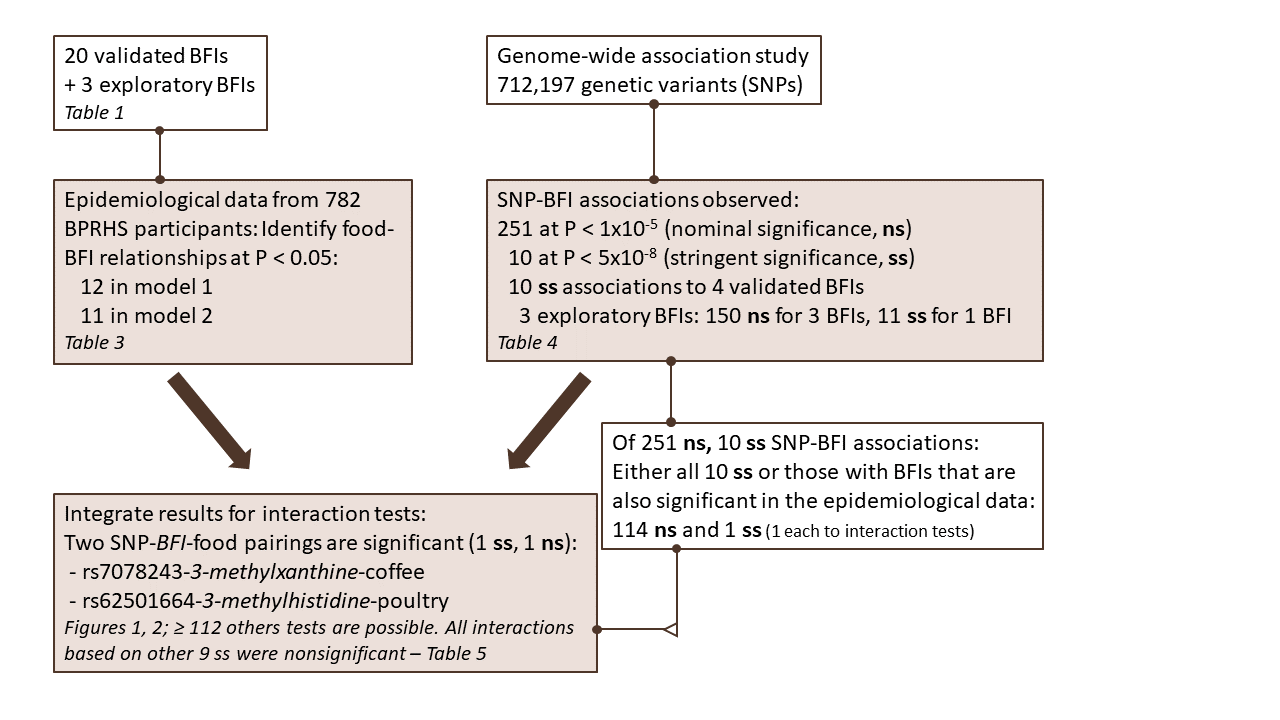


**Supplemental Figure 1. Schematic illustration of the workflow: data inputs and analytical processes.** Data input steps and data filtering are shown in boxes with white background. Analysis steps are presented in shaded boxes. References to tables and figures connect with the respective details in the manuscript.
